# Early changes in the central vision and the macula in diabetic retinal disease: associations with serum biomarkers

**DOI:** 10.3389/fendo.2026.1972307

**Published:** 2026-10-06

**Authors:** Norihiro Nagai, Hajime Shinoda, Shu Meguro, Shozo Sonoda, Taiji Sakamoto, Kazuno Negishi, Yoko Ozawa

**Affiliations:** 1 Department of Ophthalmology, Keio University School of Medicine, Tokyo, Japan; 2 Division of Nephrology, Endocrinology, and Metabolism, Department of Internal Medicine, Keio University School of Medicine, Tokyo, Japan; 3 Department of Ophthalmology, Kagoshima University Graduate School of Medical and Dental Sciences, Kagoshima, Japan; 4 Department of Clinical Regenerative Medicine, Fujita Medical Innovation Center Tokyo & Eye Center, Fujita Health University Haneda Clinic, Tokyo, Japan

**Keywords:** biomarker, diabetes, diabetic retinal disease, macula, visual acuity

## Abstract

**Background:**

Recent progress in medical science allows patients with diabetes to expect preservation of good central vision. However, the influence of diabetes-related systemic factors on the macula is not fully understood. We therefore investigated associations among central vision, macular neural and vascular parameters, and systemic biomarkers to explore the pathogenesis of early diabetic retinal disease (DRD).

**Methods:**

In this retrospective observational study, we evaluated best-corrected visual acuity (BCVA), size parameters of components of the neural retina, and blood flow in the retina and choroid in patients with diabetes and no obvious macular lesions. Optical coherence tomography (OCT) and OCT angiography (OCTA) were used for the ocular assessments. Serum biomarker levels were determined from routine blood tests. Associations among ocular parameters and between ocular parameters and serum biomarkers were analyzed using Spearman’s rank correlation coefficient analyses and multiple regression analyses adjusted for age, sex and duration of diabetes.

**Results:**

The study included 121 eyes of 121 patients (mean age, 61.7 years), of whom 80 (66.1%) were male. In analyses adjusted for age, sex and duration of diabetes, worse BCVA was associated with shorter photoreceptor outer segment (PROS) length (β = −0.278, P = 0.005) and lower retinal vessel density (β = −0.328, P = 0.002). PROS length was positively associated with retinal pigment epithelium (RPE) volume (β = 0.318, P < 0.001). RPE volume was positively associated with serum high-density lipoprotein cholesterol (HDL) levels and negatively associated with oxidized low-density lipoprotein cholesterol (LDL), uric acid and hemoglobin A1c (HbA1c) levels (β = 0.289, -0.216 −0.210, and −0.215, respectively; all P ≤ 0.05). Lower retinal and/or choroidal vascular values were associated with higher HbA1c and high-sensitivity C-reactive protein (hsCRP) levels (β = −0.256 to −0.384; all P ≤ 0.05).

**Conclusion:**

This study links subtle macular neural and vascular changes with systemic biomarkers in diabetic patients without visible macular lesions. Impaired central vision was associated with shorter PROS, lower RPE volume, and reduced vascular parameters. These findings improve understanding of early DRD and suggest that monitoring HbA1c, HDL, oxidized LDL, uric acid, and hsCRP may help estimate the risk of visual impairment.

## Introduction

Recent progress in medical science allows patients with diabetes to expect a daily life comparable to that of healthy individuals. Progression to proliferative diabetic retinopathy (PDR), a blinding complication, has been substantially reduced (1) and effective treatments for diabetic macular edema (DME) have been established. Therefore, the next challenge is not merely to prevent blindness but to preserve or restore good central vision and visual acuity. Central visual function depends on the condition of the macula, the central part of the neural retina. However, the early pathogenesis of macular neural tissue in diabetes—particularly with no pronounced secondary alterations, such as those caused by PDR or DME—is not well understood and its associations with serum, and systemic biomarkers are not well investigated.

Diabetic retinopathy (DR) has long been assessed primarily as a microangiopathy. However, recent studies have proposed the concept of diabetic retinal disease (DRD), which recognizes that retinal pathology involves not only the vascular system but also neural and glial cells and their interactions within the neurovascular unit (2, 3). This concept has increased interest in preclinical and early clinical signs of DRD to better understand the mechanisms and timing of disease onset (2). Both basic and clinical studies have demonstrated subtle but clear functional and structural changes in retinal neural tissue in diabetes (4–8). To analyze these early changes, we have been studying diabetic eyes at a stage before visible macular changes are detectable on fundus photographs and before the development of secondary changes associated with PDR and/or exudative fluid in DME (9, 10). However, their associations with systemic biomarkers have not yet been addressed, although diabetic complications tend to be associated not only with hyperglycemia but also with broader systemic metabolic alterations.

Non-invasive ocular imaging systems, such as optical coherence tomography (OCT) and OCT angiography (OCTA), can detect subtle changes at the micrometer scale and are now widely used in clinical practice. OCT enables measurement not only of the thickness of the neural retinal layers but also of photoreceptor outer segment (PROS) length. Length of the PROS, where visual pigments are concentrated and act as light receptors, has been reported to be reduced in eyes with diabetes than in control (10), and as a marker of photoreceptor condition in eyes at high risk of age-related macular degeneration (11, 12). The volume of the retinal pigment epithelium (RPE), a highly metabolic single-cell layer essential for photoreceptor maintenance, can also be measured on OCT images.

In this study, we investigated patients with diabetes but no obvious macular lesions on fundus photographs. Using OCT and OCTA, we measured retinal structure and capillary blood flow and examined their relationships with serum levels of biomarkers which were obtained from routinely performed blood tests. We hope this will improve understanding of early retinal changes in diabetes and systemic factors associated with them.

## Materials and methods

This study adhered to the tenets of the Declaration of Helsinki and was approved by the Ethics Committee of Keio University School of Medicine (approval number: 20100003). The need for written informed consent was waived, and an opt-out approach was used.

### Participants

Patients with diabetes who visited the Division of Diabetes, Department of Internal Medicine, Keio University Hospital (Tokyo, Japan), and were referred to the Department of Ophthalmology between July 2024 and July 2025 for screening of DR were included. Patients who reported subjectively good visual acuity were included. Data from the right eye of each patient were included unless the right eye met an exclusion criterion, in which case data from the left eye were included. The exclusion criteria were: visible diabetic lesions in the macular area, such as DME; moderate non-proliferative DR or more severe DR; or other retinal diseases such as retinal vein occlusion, epiretinal membrane, age-related macular degeneration, high myopia with axial length ≥26.5 mm, or glaucoma. Eyes with best-corrected visual acuity (BCVA) > 0.155 logarithm of the minimum angle of resolution (logMAR) (equivalent to 0.7 on the decimal score, as measured using a Landolt C chart) were also excluded.

### Eye examinations

The patients underwent ophthalmic examinations, including measurement of BCVA, intraocular pressure (IOP), and axial length, and acquisition of fundus photographs (California, Optos, Inc., MA), sectional and three-dimensional spectral-domain OCT images (Spectralis OCT, Heidelberg Engineering GmbH, Dossenheim, Germany), and 3 x 3 mm OCT angiography (OCTA) (OCT HS-100; Canon, Inc., Tokyo, Japan) images. For the analyses for CC, OCTA images were split into 13 × 13 smaller images with built-in software and the central 5x5 images (about 1.15 mm square) were analyzed. Central retinal thickness (CRT), central choroidal thickness (CCT), and vessel density in the retinal superficial capillary plexus (SCP) and choriocapillaris (CC) were measured using built-in calipers and proprietary software provided with the devices. For the vessel density measurement of SCP and CC, automatic adjustment of axial length was involved in the built-in software. The choroidal luminal/stromal ratio in the central 3,000 μm area was analyzed using Kago-Eye 2 software established by Sonoda et al. as described previously (13–16).

### Systemic tests

Blood tests and blood pressure measurements were ordered by the Department of Internal Medicine, Keio University, and performed at Keio University Hospital. Blood samples were collected under fasting conditions as part of routine clinical testing. The blood test performed nearest to and before the ophthalmic examination was used, with a maximum interval of three months.

### Statistical analysis

IBM SPSS Statistics (version 31.0; IBM Corp., Armonk, NY, USA) was used for all statistical analyses. All data are presented as means ± standard deviations. Spearman’s rank correlation coefficient analysis and multiple regression analysis were used to analyze the data. Statistical significance was set at P < 0.05.

## Results

### Clinical characteristics of the participants and included eyes

The mean age of the 121 patients was 61.7 ± 13.5 years (range, 24–89; median, 62) (**Table 1**). Eighty patients (66.1%) were male, and the mean duration since diabetes diagnosis was 10.7 ± 10.1 years (0–50, 9). The mean HbA1c level was 7.5 ± 1.4% (5.5–13.3; 7.1). Blood test and blood pressure data are shown in **Table 1**.

**Table 1:** Characteristics of the patients.

Factors	Value
Age	61.7 ± 13.5 (24–89, 62)
Sex (Male %)	80 (66.1%)
Duration of diabetes (years)	10.7 ± 10.1 (0–50, 9)
HbA1c (%)	7.5 ± 1.4 (5.5-13.3, 7.1)
Creatinine (μmol/L)	73.2 ± 25.4 (29.2-247.5, 69.0)
eGFR (mL/min/1.73m^2^)	71.2 ± 17.6 (18.0-115.0, 70.0)
Total cholesterol (mmol/L)	5.01 ± 0.94 (2.64-7.78, 4.97)
Triglyceride (mmol/L)	1.78 ± 1.25 (0.32-6.88, 1.40)
High-density lipoprotein cholesterol (HDL, mmol/L)	1.40 ± 0.44 (0.78-2.97, 1.27)
Low-density lipoprotein cholesterol (LDL, mmol/L)	2.85 ± 0.84 (1.19-6.05, 2.91)
Oxidized LDL (U/L)	103 ± 45 (35-318, 96)
High sensitivity C-reactive protein (mg/dL)	0.27 ± 0.69 (0.01-5.81, 0.10)
Uric acid (μmol/L)	318 ± 85 (125-553, 309)
WBC (×10^9^/L)	6.83 ± 1.99 (3.10-12.30, 6.50)
RBC (×10^12^/L)	4.76 ± 0.55 (2.68-6.11, 4.81)
Hemoglobin (g/dL)	14.6 ± 1.5 (10.6-19.1, 14.6)
Systolic blood pressure (SBP, mmHg)	140 ± 17 (104-195, 137)
Diastolic blood pressure (DBP, mmHg)	83 ± 13 (46-121, 82)
Pulse pressure (mmHg)	58 ± 16 (19-108, 54)

Data are presented as mean ± standard deviation (range, median). HbA1c, Hemoglobin A1c; eGFR, estimated glomerular filtration rate; WBC, white blood cell; RBC, red blood cell.

All 121 included eyes exhibited no or minimal DR and had no visible macular lesions, such as DME, obvious areas of nonperfusion, fibrovascular proliferation, or neovascularization, on fundus photographs. The mean BCVA was -0.053 ± 0.057 logMAR (-0.079 to 0.155; -0.079); a logMAR value of -0.079 corresponds to 1.2 on the Landolt C decimal score (25/20 Snellen equivalent), whereas 0.155 corresponds to 0.7 on the decimal scale, which is the minimum visual acuity requirement for a driver’s license in Japan. The mean intraocular pressure (IOP) was 15.8 ± 2.8 mmHg (9.0–21.0; 16.0), and within the normal range. Data on retinal and choroidal structures recorded by OCT and OCTA are also shown in **Table 2**.

**Table 2:** Characteristics of the eyes.

Factors	Value
Best-corrected visual acuity (BCVA, logMAR)	-0.053 ± 0.057 (-0.079-0.155, -0.079)
Intraocular pressure (IOP, mmHg)	15.8 ± 2.8 (9.0-21.0, 16.0)
Axial length (mm)	24.3 ± 1.1 (20.9-26.5, 24.2)
Photoreceptor outer segment (PROS) length (μm)	57.6 ± 6.4 (41-75, 58)
Central retinal thickness (CRT, μm)	233 ± 22 (174-292, 232)
Central choroidal thickness (CCT, μm)	235 ± 103 (62-588, 228)
Volume of the whole retina (mm^3^)	8.50 ± 0.41 (7.69-9.66, 8.50)
Volume of retinal pigment epithelium (RPE, mm^3^)	0.38 ± 0.03 (0.32-0.46, 0.39)
Vessel density in superficial capillary plexus (SCP, %)	37.2 ± 2.6 (26.4-41.1, 37.9)
Vessel density in choriocapillaris (CC, %)	45.1 ± 0.8 (42.8-48.0, 45.0)
Choroidal luminal/stromal ratio	2.95 ± 1.22 (1.44-9.34, 2.55)

Data are presented as mean ± standard deviation (range, median).

### Associations of best-corrected visual acuity (BCVA) with retinal and choroidal parameters

Although none of the included eyes had visible retinal lesions in the macular area, their central visual function, evaluated by BCVA, varied and was correlated with structural changes in the retina and choroid measured by OCT and OCTA (**Figure 1**). BCVA in logMAR was significantly correlated with the length of the photoreceptor outer segments (PROS)——with shorter PROS indicating worse BCVA (**Figures 1A**, **2**; ρ = -0.308, P = 0.001). In contrast, retinal ganglion cell layer (GCL) volume was not significantly correlated with BCVA (**Figure 1B**; ρ = -0.158, P = 0.087).

**Figure 1:**
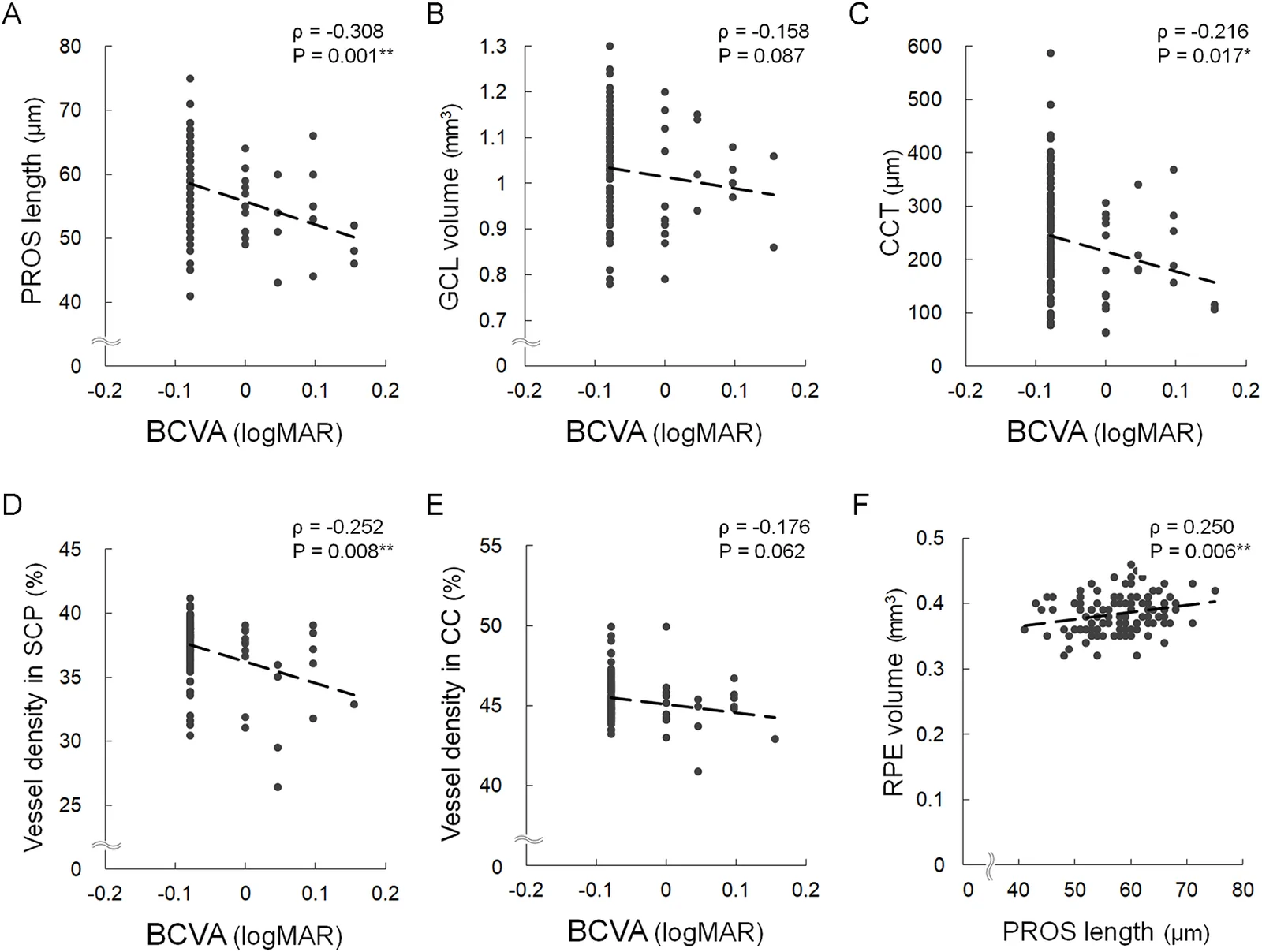
Correlations of best-corrected visual acuity (BCVA) and photoreceptor outer segment (PROS) length with other ocular parameters. Correlations between BCVA and PROS length **(A)**, retinal volume of ganglion cell layer (GCL) **(B)**, central choroidal thickness (CCT) **(C)**, vessel flow density in superficial capillary plexus (SCP) **(D)**, and vessel flow density in choriocapillaris (CC) **(E)**, and between PROS length and retinal pigment epithelium (RPE) volume **(F)**, are shown. Spearman’s rank correlation coefficient analyses were performed. *P < 0.05, **P < 0.01.

**Figure 2:**
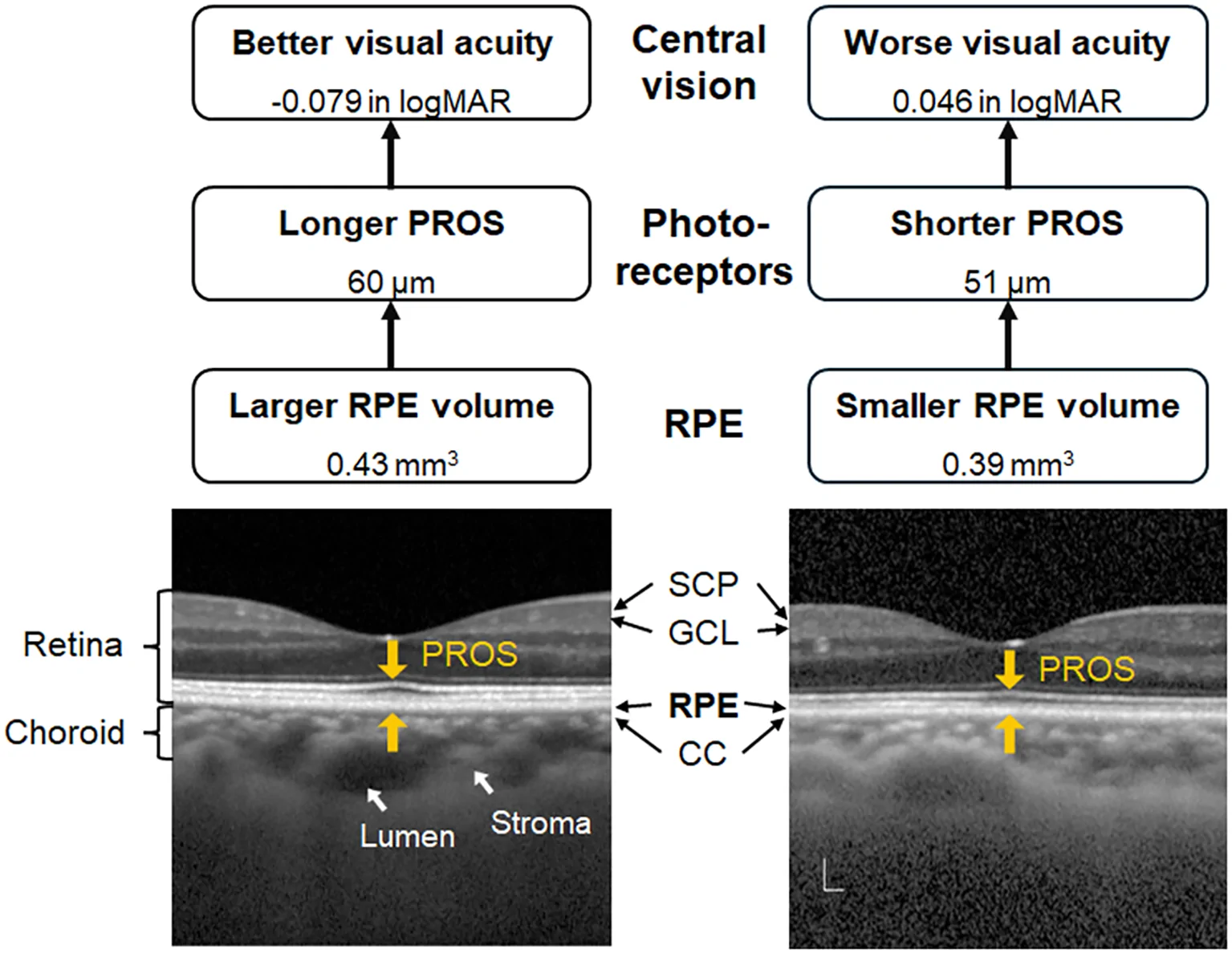
Representative sectional optical coherence tomography images of the retina and choroid in eyes with relatively better and worse best-corrected visual acuity (BCVA). The upper and lower boundaries of the photoreceptor outer segment (PROS) are indicated by yellow arrows. Annotations are included to aid interpretation. The luminal and stromal components of the choroid adjacent to the retinal pigment epithelium (RPE) are indicated by white arrows. GCL, ganglion cell layer; SCP, superficial capillary plexus; CC, choriocapillaris.

Better vision (lower logMAR BCVA) was also correlated with a thicker choroid (**Figure 1C**; ρ = -0.216, P = 0.017), a structure that nourishes the outer retinal layers including photoreceptor cells, greater vessel flow density in the retinal superficial capillary plexus (SCP) which is supplied by the central retinal artery (**Figure 1D**; ρ = -0.252, P = 0.008). There was a trend toward a correlation between the logMAR BCVA and vessel flow density in the choriocapillaris (CC), the choroidal capillary network which is supplied by the ciliary arteries, (**Figure 1E**; ρ = -0.176, P = 0.062). Representative images of eyes with better vision and greater vessel flow densities in the SCP and CC, and eyes with worse vision and lower vessel flow densities in the SCP and CC are shown in **Figure 3**.

**Figure 3:**
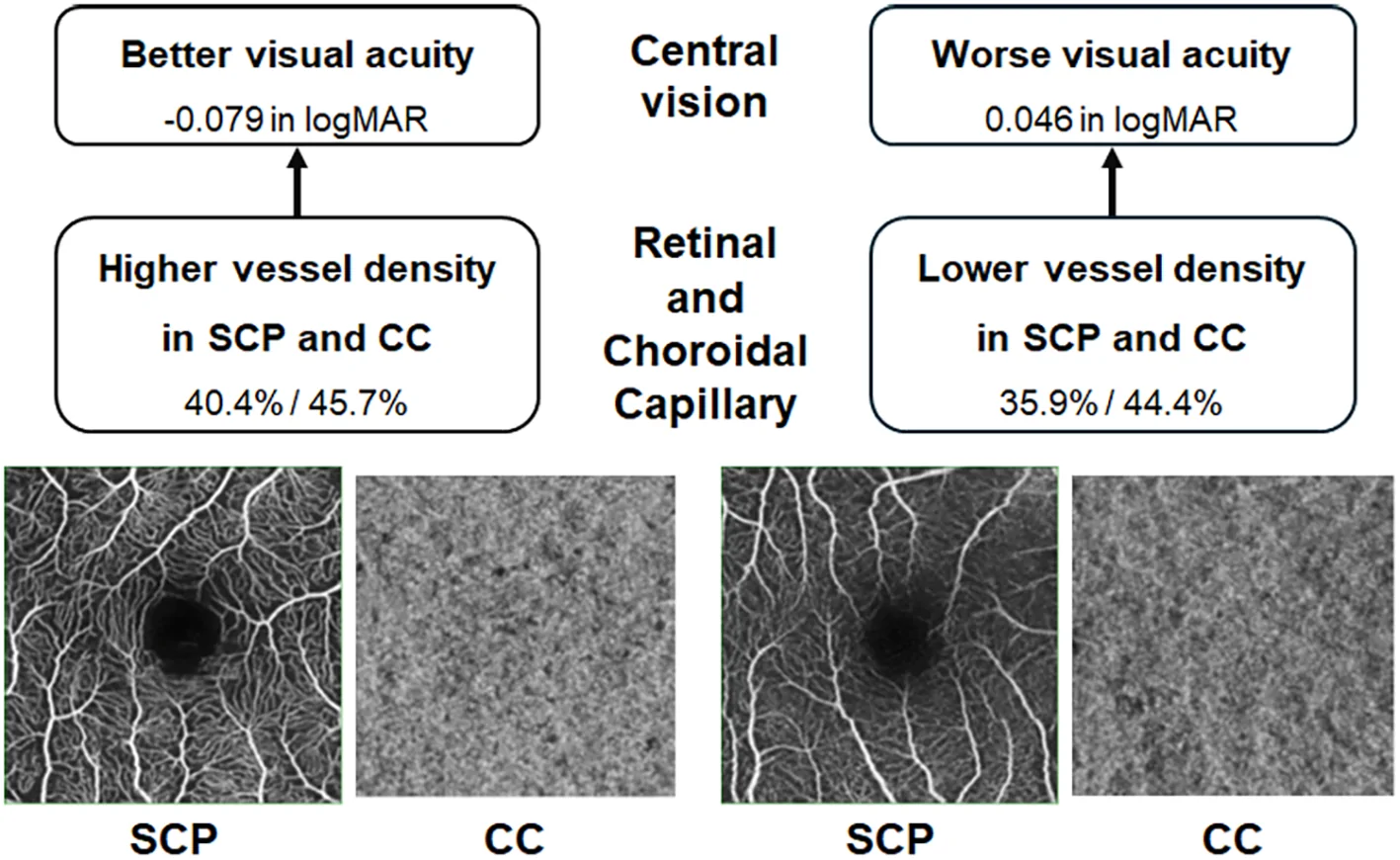
Representative optical coherence tomography angiography images of retinal and choroidal blood flow in eyes with relatively better and worse best-corrected visual acuity (BCVA). Vessel flow densities in the retinal superficial capillary plexus (SCP) and choriocapillaris (CC), measured using three-dimensional recording of optical coherence tomography (OCT) angiography, are shown.

### Associations among retinal and choroidal parameters

Greater RPE volume was associated with longer PROS (**Figure 1F**; ρ = 0.250, P = 0.006). The RPE plays a role in maintaining photoreceptor homeostasis and transporting nutrients from the choroidal vascular network to photoreceptor cells (17, 18). Thus, atrophic changes in the RPE may have negatively affected PROS. Representative images of eyes with better vision with longer PROS and larger RPE volume, and eyes with worse vision, shorter PROS, and smaller RPE volume are shown in **Figure 2**.

Correlations among the ocular parameters were then analyzed using multiple regression analyses adjusted for age, sex and duration of diabetes (**Table 3**). The correlation between BCVA and PROS length remained significant after adjustment (β = -0.279, P = 0.005). BCVA was also correlated with vessel flow density in the SCP (β = -0.328, P = 0.002), and there was a trend toward a correlation between BCVA and vessel flow density in the CC (β = -0.180, P = 0.051). The correlation between PROS length and RPE volume also remained significant after adjustment (β = 0.318, P < 0.001).

**Table 3:** Correlations between ocular parameters.

Characteristic	Parameters	β	P	95%CI
BCVA (logMAR)	PROS length	-0.279	0.005**	-0.004 - -0.001
	RGC volume	-0.027	0.782	-0.001 - 0.001
	CCT	-0.161	0.082	0.000 - 0.000
	Vessel density in SCP	-0.328	0.002**	-0.010 - -0.003
	Vessel density in CC	-0.180	0.051	-0.013 - 0.000
PROS length	RPE volume	0.318	<0.001**	0.364 - 1.079
	Choroidal L/S ratio	0.133	0.109	-0.159 - 1.556
	Vessel density in SCP	0.084	0.389	-0.256 - 0.653
Volume of RPE	Choroidal L/S ratio	0.270	0.003**	0.220 - 1.032
Choroidal L/S ratio	CCT	0.399	<0.001**	0.003 - 0.007
	Vessel density in SCP	0.189	0.075	-0.009 - 0.185
Vessel density in SCP	CCT	0.156	0.086	-0.001 - 0.008
	Vessel density in CC	0.348	<0.001**	0.341 - 0.963

Multiple regression analysis for ocular parameters adjusted with age, sex and duration of diabetes. BCVA, best-corrected visual acuity; MAR, Minimum Angle of Resolution; CCT, central choroidal thickness, PROS, photoreceptor outer segment; SCP, superficial capillary plexus; CC, choriocapillaris; RPE, retinal pigment epithelium; Choroidal L/S ratio, choroidal luminal/stromal ratio. ** P<0.01.

RPE volume was correlated with the choroidal luminal/stromal (L/S) ratio (β = 0.270, P = 0.003), and the ratio was correlated with CCT (β = 0.399, P < 0.001), indicating that RPE volume was smaller when the choroidal vascular luminal ratio and flow area were smaller; these findings also corresponded to a thinner choroid. Vessel flow densities in the SCP and CC were positively correlated with each other (β = 0.348, P < 0.001).

### Associations between systemic biomarkers and ocular parameters

Next, relationships between systemic data and ocular parameters were analyzed using multiple regression analyses adjusted for age, sex and duration of diabetes (**Table 4**). RPE volume was positively correlated with serum levels of high-density lipoprotein cholesterol (HDL) (β = 0.289, P = 0.002) and negatively correlated with serum levels of oxidized low-density lipoprotein cholesterol (LDL) (β = -0.216, P = 0.040), uric acid (β = -0.210, P = 0.022). There was a trend toward a negative correlation between RPE volume and WBC counts (β = -0.183, P = 0.050). The RPE volume was also negatively correlated with serum levels of HbA1c (R = -0.215, P = 0.018). HbA1c was also negatively correlated with ocular vascular parameters: the choroidal L/S ratio (β = -0.187, P = 0.042) and vessel flow density in the SCP (β = -0.384, P < 0.001). High-sensitivity C-reactive protein (hsCRP) levels were negatively correlated with vessel flow density in the SCP (β = -0.329, P < 0.001) and the CC (β = -0.256, P = 0.011).

**Table 4:** Correlations between ocular and systemic parameters.

Characteristic	Parameters	β	P	95%CI
Volume of RPE	Triglyceride	-0.177	0.054	-0.009 - 0.000
	HDL	0.289	0.002**	0.018 - 0.078
	Uric acid	-0.210	0.022*	-0.780 - -0.061
	Oxidized LDL	-0.216	0.040*	-0.027 - -0.001
	HbA1c	-0.215	0.018*	-0.772 - -0.074
	WBC	-0.183	0.050	-0.519 - 0.000
Vessel density in SCP	HbA1c	-0.384	<0.001**	-0.958 - -0.386
	hsCRP	-0.329	<0.001**	-1.797 - -0.546
Vessel density in CC	hsCRP	-0.256	0.011*	-0.902 - -0.119
Choroidal L/S ratio	HbA1c	-0.187	0.042*	-0.310 - -0.006

Multiple regression analysis for ocular parameters adjusted with age, sex and duration of diabetes. RPE, retinal pigment epithelium; HDL, High-density lipoprotein cholesterol; LDL, Low-density lipoprotein cholesterol; HbA1c, Hemoglobin A1c; hsCRP, high sensitivity C-reactive protein; WBC, white blood cell; Choroidal L/S ratio, choroidal luminal/stromal ratio; SCP, superficial capillary plexus; CC, choriocapillaris. * P<0.05, ** P<0.01.

## Discussion

The expanding concept of DRD recognizes that neural and vascular alterations may develop before the appearance of the retinal lesions traditionally used to diagnose DR. In this study, we examined relationships among systemic biomarkers, macular neural and vascular parameters, and BCVA in diabetic patients with relatively good vision and no obvious macular lesions as well as no PDR or DME (**Figure 4**). We found that central vision, evaluated by BCVA, was associated with ocular parameters, including PROS length and retinal vessel flow density in the SCP, with a trend toward an association with choriocapillaris flow density (**Figure 1**, **Table 3**). Condition of the photoreceptor was well correlated with that of the RPE volume (**Figure 1**, **Table 3**). As a novel finding in diabetic patients, systemic biomarkers reflecting lipid metabolism, uric acid metabolism, and inflammation, as well as glycemic control (HDL, oxidized LDL, uric acid, WBC count, and hsCRP, HbA1c) were associated with early neural and vascular alterations in the macula (**Table 4**). These findings suggest the relevance of systemic metabolic and inflammatory status, not just of glycemic control, to early DRD.

**Figure 4:**
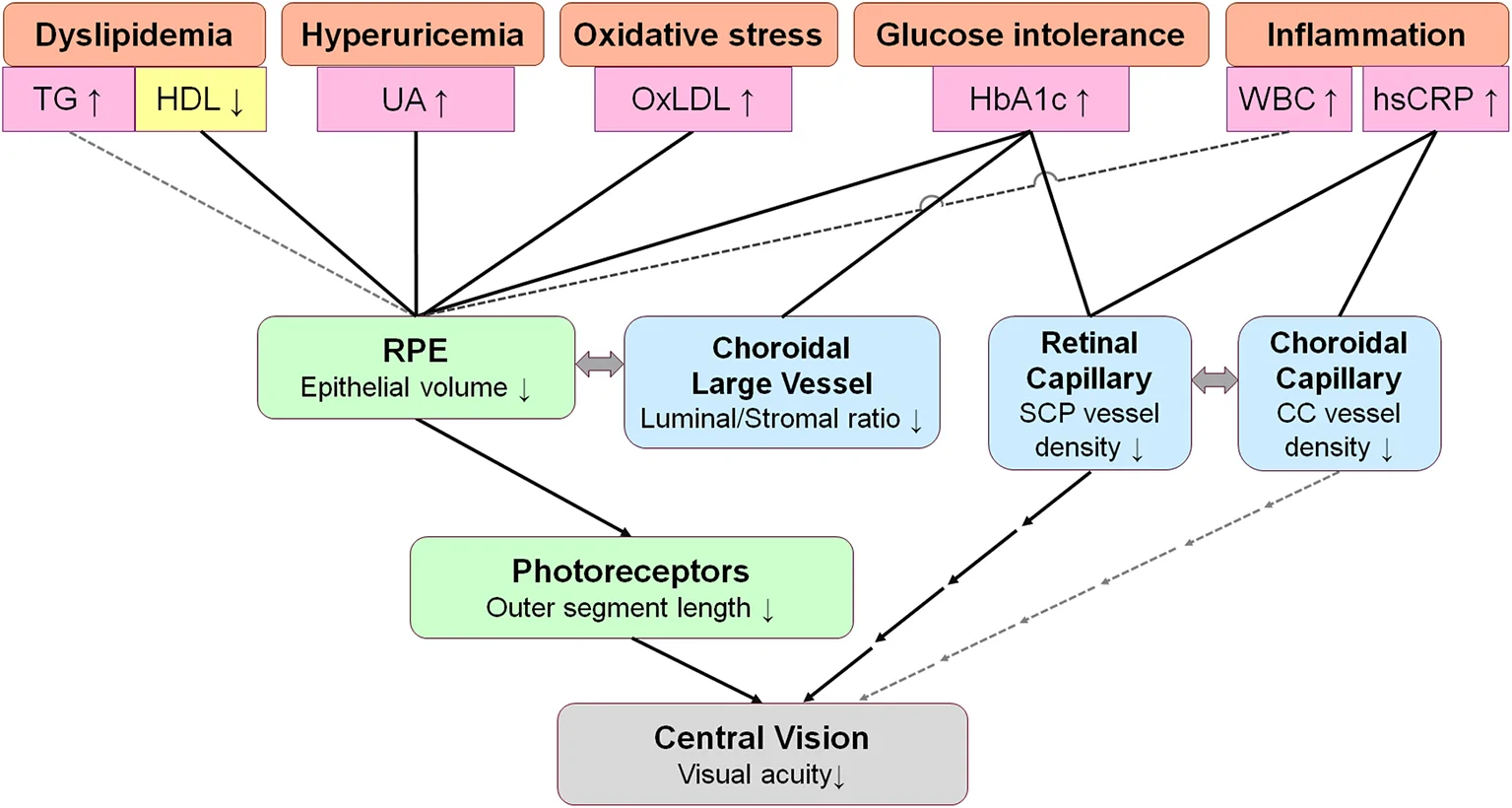
Schematic summary of the correlations among central vision, neural and vascular parameters of the retina and choroid, and serum biomarkers. Serum, and systemic biomarkers are grouped according to the processes they represent. Solid lines and arrows indicate significant correlations, whereas dashed lines and arrows indicate trends toward correlations.

### Central vision and photoreceptor changes

Although none of the patients had macular lesions obvious on fundus photographs, BCVA varied with PROS length. Because mice do not have a macula and their central vision cannot be measured, this relationship could only be demonstrated using human data.

Most previous basic studies have focused on degeneration of the retinal GCL as a representative form of neurodegeneration in diabetes (4, 5). However, GCL volume was not associated with central vision in the current study. This is consistent with the fact that GCL degeneration, a characteristic finding in glaucoma, primarily causes visual field defects rather than impairments of central vision.

Central vision directly affects daily life (19), and our finding that worse BCVA was associated with shorter PROS is clinically relevant. Importantly, because photoreceptor outer segments are phagocytosed by the RPE and renewed daily (20), their shortening may be reversible if the condition of the photoreceptor cells improves. Further studies are warranted.

### Retinal and choroidal vascular changes

Vessel flow density in the retinal SCP was also associated with BCVA. The retinal circulation primarily supplies oxygen and nutrients to the inner retinal layers, including GCL, and not the photoreceptor cell bodies which compose of outer retina. However, photoreceptor synapses with second-order neurons are located in the outer plexiform layer, at the innermost part of the outer retina, and are therefore also supplied by the retinal circulation. Lower SCP vessel flow density may reflect reduced overall retinal flow and reduced oxygen and nutrient supply to photoreceptor synapses, which could contribute to worse central vision.

The retinal and choroidal vascular systems, represented here by the SCP and CC, respectively, both originate from the carotid circulation and are affected by blood pressure. However, their local regulation differs. Retinal blood flow is mainly regulated by hypoxia (16), whereas choroidal blood flow is regulated by the autonomic nervous system, particularly under disease conditions (17, 18). Nonetheless, vessel flow densities in the SCP and CC were positively correlated (β = 0.341, P < 0.001).

### Metabolic changes and retinal pigment epithelium volume

The present study found that RPE volume was positively correlated with PROS length and was also correlated, in different directions, with several systemic biomarkers. The RPE is a highly metabolic tissue that is essential for photoreceptor maintenance (17, 18) and takes up circulating lipids (21, 22). The effects of lipid metabolism on the RPE have been studied in animal models. Under conditions of excessive lipid loading, RPE lipid metabolism may become impaired, and metabolically exhausted RPE cells may produce abnormal metabolites that contribute to chronic inflammatory diseases such as age-related macular degeneration (21–23). In the current study, lower serum HDL levels were correlated with lower RPE volume. Given that HDL reportedly regulates lipid efflux from the RPE (24), insufficient HDL may impair lipid metabolism in the RPE. Oxidized LDL, which is produced by the oxidative modification of LDL, is a biomarker of oxidative stress (25), and can promote vascular and neural damage by activating macrophages (26). The results of animal experiments showing that the RPE degeneration was related to overloaded oxidized LDL to the macrophages in mice constantly fed high fat diet was consistent with the current result (23).

Animal experiments have shown that hyperuricemia can reduce gap junction protein expression and increase apoptosis in the RPE (27). The same study reported protective effects of the uric acid-lowering agents allopurinol and benzbromarone on the RPE. One of the important roles of the RPE is to form the outer blood-retinal barrier, and disruption of this barrier may impair nutrient transport to photoreceptors. High serum uric acid levels may adversely affect the RPE and ultimately contribute to a loss of quality of central vision.

### Inflammation, glycemic control, and vascular impairment

Lower vessel flow densities in the retinal and choroidal capillaries were correlated with higher hsCRP levels. Although animal experiments have demonstrated inflammatory changes in the diabetic retina (28, 29), hsCRP is not used routinely to estimate the risk of DRD progression in clinical practice. However, hsCRP measurement may be valuable for evaluating inflammation, especially in relation to diabetic microangiopathy. Even when HbA1c has been improved by treatment, if tissues and organs previously exposed to poor glycemic control remain inflamed—as represented by high levels of hsCRP—this may secondarily affect the retinal and choroidal vasculature. HbA1c was associated with RPE volume, the choroidal L/S ratio, and vessel flow density in the SCP. Clinicians routinely consider HbA1c when estimating the risk of diabetic complications, and adequate glycemic control remains indispensable for preventing DR progression. The concept is based on the fact that HbA1c is associated with diabetic complications, in particular, related to microangiopathy which had been assessed as the main pathogenesis in the retina with diabetes (30, 31). However, the present results suggest that different systemic biomarkers may reflect different aspects of early DRD: lipid and uric acid levels were associated mainly with RPE volume, whereas hsCRP was associated with retinal and choroidal capillary flow. Considering these biomarkers in addition to HbA1c may therefore provide a broader early assessment of subtle neural and vascular alterations related to central vision. Such an assessment may become increasingly important as modern patients expect not only prevention of severe visual loss but also preservation of high-quality central vision.

## Limitations

This study has several limitations, including its relatively small sample size, retrospective and cross-sectional design, and the absence of an assessment of smoking and medication histories, and body mass index. The analyses showed correlations but not causality. Nevertheless, the findings identified ocular and systemic parameters that may deserve greater attention in clinical practice and may be useful to include in future studies of neuroprotective approaches aimed at preserving central vision.

## Conclusion

In diabetic patients without macular lesions visible on fundus photographs, worse central vision was associated with shorter PROS and lower retinal vessel flow density. RPE volume and retinal and choroidal vascular parameters were also associated with serum biomarkers, including HbA1c, lipid levels, uric acid, and hsCRP. These findings indicate that early DRD involves interconnected neural, vascular, and systemic alterations. Broader assessment of these biomarkers may help identify patients at risk of subtle and potentially reversible macular changes and support the development of neurovascular unit-protective approaches aimed at preserving high-quality central vision. Further studies are warranted.

## Data Availability

The datasets generated for this study are available on request to the corresponding author.
